# Optimizing Blood Pressure Management in Type 2 Diabetes: A Comparative Investigation of One-Time Versus Periodic Lifestyle Modification Counseling

**DOI:** 10.7759/cureus.61607

**Published:** 2024-06-03

**Authors:** Shweta Malakar, Shivendra Kumar Singh, Kauser Usman

**Affiliations:** 1 Community Medicine, King George’s Medical University (KGMU), Lucknow, IND; 2 Internal Medicine, King George’s Medical University (KGMU), Lucknow, IND

**Keywords:** glycemic management, periodic counseling, comparative investigation, cardiovascular risk, randomized controlled trial, hypertension, counseling, lifestyle modification, blood pressure management, type 2 diabetes

## Abstract

Background

Type 2 diabetes mellitus (T2DM) often coexists with hypertension, significantly increasing cardiovascular risks. Lifestyle modification counseling has shown promise in managing T2DM and its comorbidities. However, the optimal frequency and structure of counseling for blood pressure control remain uncertain. Our study examines the best approach for managing blood pressure in T2DM patients by comparing the outcomes of two counseling strategies: a single session and periodic counseling over time.

Methodology

A total of 110 diabetic patients were enrolled, with 52 patients in each group after loss to follow-up. A randomized controlled trial compared one-time counseling (control) to six months of periodic counseling (intervention) on lifestyle modification. A weighing machine, stadiometer, 24-hour dietary recall, food frequency questionnaire, biochemical blood sugar level analysis, and telephonic follow-up were the essential tools used. The data were analyzed using SPSS version 24.0 (IBM Corp., Armonk, NY, USA), employing descriptive statistics, including frequencies, percentages, graphs, mean, and standard deviation. Statistical significance at the 5% level was tested using probability (p) calculations. The Kolmogorov-Smirnov test confirmed normal distribution (p > 0.05). Parametric tests, specifically independent t-tests, were used for between-group comparisons of continuous variables, while categorical variables were analyzed using the chi-square test or Fisher’s exact test. Intragroup comparisons over time employed repeated-measures analysis of variance for continuous variables. Changes within groups after six months were assessed using paired t-tests. All statistical analyses adhered to a significance level of p < 0.05.

Results

The gender distribution at baseline was similar between the control (55.8% male, 44.2% female) and intervention (46.2% male, 53.8% female) groups, with no significant differences (p = 0.327). The mean weight was 66.67 ± 11.51 kg in the control group and 67.14 ± 11.19 kg in the intervention group (p = 0.835), and the body mass index was 25.61 ± 4.09 kg/m² and 26.29 ± 6.01 kg/m², respectively (p = 0.503). Clinical parameters such as fasting blood sugar, postprandial blood sugar, glycosylated hemoglobin, and blood pressure showed no significant differences between the control and intervention groups at baseline (p > 0.05). After six months, the intervention group exhibited a trend toward lower blood pressure compared to the control group, but the differences were not statistically significant. The mean systolic blood pressure was 132.15 ± 14.867 mmHg in the control group and 129.15 ± 9.123 mmHg in the intervention group (p = 0.218). Changes in blood pressure over the six-month period showed significant decreases within the intervention group, while changes in the control group did not reach statistical significance. The mean difference in systolic blood pressure in the intervention group was 5.54 ± 9.77 mmHg (p = 0.0001), indicating a notable reduction, while the control group had a smaller and statistically insignificant increase of 2.308 ± 9.388 mmHg (p = 0.082).

Conclusions

This study addresses a significant gap in the literature by comparing the efficacy of one-time vs. periodic counseling in T2DM management. While periodic counseling shows promise in improving diastolic blood pressure, further research is needed to understand its nuanced effects and optimize lifestyle interventions for T2DM patients.

## Introduction

In recent years, diabetes and hypertension have become increasingly prevalent health concerns globally, with significant implications for public health and healthcare systems. According to the International Diabetes Federation, in 2021, approximately 537 million adults (aged 20-79 years) worldwide were living with diabetes [1]. This figure represents a 16% increase from the 2019 data, highlighting the rapid growth of the diabetes epidemic. Projections indicate that by 2030, the number of people with diabetes is expected to reach 643 million, with further growth to 783 million by 2045. People with type 2 diabetes mellitus (T2DM) frequently experience a wide range of problems, one of which is hypertension, which dramatically raises the risk of cardiovascular events and mortality [2]. Two-thirds of patients with T2DM have arterial hypertension [3]. As hypertension is a prevalent comorbidity in T2DM, controlling it well is essential to reducing the cardiovascular risks associated with it.

To effectively manage T2DM and its related comorbidities, such as hypertension, lifestyle modification, which includes dietary adjustments, increased physical activity, and weight management, remains essential. Counseling programs that support these lifestyle changes have demonstrated the potential to lower cardiovascular risk factors and enhance glycemic management [4].

However, further research is required to ascertain the optimal frequency and format of counseling for lifestyle modifications aimed at controlling blood pressure in patients with T2DM. This study aims to advance this discussion by comparing the effectiveness of single-session lifestyle change counseling with continuous counseling sessions in enhancing blood pressure management among individuals with T2DM.

Although other studies have demonstrated the beneficial effects of lifestyle change on blood pressure control [5-8], there is still a lack of information about the relative efficacy of recurring interventions versus one-time counseling. Our study aims to give evidence-based insights into the most effective way to optimize blood pressure management in T2DM by carefully evaluating these two counseling modalities.

Comprehending the subtle impacts of different counseling approaches is essential to customizing interventions to the specific requirements of each patient, ultimately improving the standard of care for those who are managing both hypertension and T2DM. In the context of T2DM, this study attempts to close a significant gap in the literature and guide medical practitioners in developing focused, patient-centered blood pressure management techniques.

## Materials and methods

Study design and setting

This study was conducted as a randomized controlled trial (RCT) with parallel group allocation. The trial was designed to be fixed with a 1:1 ratio between the experimental and control groups. The study was conducted at King George’s Medical University, Uttar Pradesh, from July 2020 to April 2022.

Ethical approval

The study protocol was approved by the Ethics Committee of King George’s Medical University, Uttar Pradesh (approval number: 93rd ECM II B-Ph.D/P2). The trial was registered with the Clinical Trials Registry-India (CTRI) under the registration number CTRI/2020/07/026780.

Participants

A total of 110 diabetic patients were enrolled in the study. Participants were recruited from the medicine outpatient department (OPD) at King George’s Medical University, Uttar Pradesh. This RCT with a parallel group allocation was conducted as a fixed trial with a 1:1 ratio between the experimental and control groups. A total of 110 diabetics were enrolled, and after accounting for loss to follow-up, 52 patients in each group, that is, control (one-time counseling on lifestyle modification) and intervention group (six months periodic counseling on lifestyle modification) were enrolled from the medicine OPD at King George’s Medical University, Uttar Pradesh. The primary outcome measure was blood pressure control among patients with T2DM.

Inclusion criteria

The inclusion criteria encompassed T2DM patients aged 30 to 65 years, currently using hypoglycemic drugs, with a glycosylated hemoglobin (HbA1c) level of 6.5% or higher, and capable of comprehending and consenting to the provided documentation.

Exclusion criteria

The exclusion criteria for this study included T2DM patients who were on insulin, pregnant and lactating women, diabetic patients with other severe comorbidities, and patients with physical immobility.

Data collection

Demographic data, biochemical data (including fasting blood sugar, postprandial sugar, and HbA1c), and clinical data (body mass index (BMI), weight, and waist-hip ratio) were collected as part of the study. Additionally, information on dietary habits, knowledge, lifestyle factors, and stress conditions was gathered. The intervention involved counseling sessions for participants covering diet, physical activity, lifestyle modifications, and disease conditions. Following the initial session, the control group was scheduled for a follow-up visit after six months, while the experimental group received periodic counseling every month for six months. To minimize dropouts and ensure adherence to the diet plan, both groups underwent periodic telephonic follow-ups conducted monthly throughout the study period.

Intervention

Participants were randomly assigned to either the control group, which received one-time counseling on lifestyle modification, or the intervention group, which received periodic counseling on lifestyle modification over six months. The intervention involved counseling sessions delivered by trained healthcare professionals. These sessions covered various aspects of diabetes management, including diet modification, physical activity promotion, lifestyle adjustments, and disease education. Participants in the intervention group received monthly counseling sessions for six months, with each session tailored to address their individual needs and challenges in managing diabetes. The counseling sessions aimed to empower participants with the knowledge and skills necessary to make sustainable lifestyle changes and improve their diabetes management outcomes.

Tools required

A weighing machine, stadiometer, 24-hour dietary recall, food frequency questionnaire, biochemical blood sugar level analysis, and telephonic follow-up were the essential tools for implementing the study.

Statistical analysis

Per-protocol analysis was used for data analysis. The data were analyzed using SPSS version 24.0 (IBM Corp., Armonk, NY, USA), employing descriptive statistics, including frequencies, percentages, graphs, mean, and standard deviation. Statistical significance at the 5% level was tested using probability (p) calculations. The Kolmogorov-Smirnov test confirmed normal distribution (p > 0.05). Parametric tests, specifically independent t-tests, were used for between-group comparisons of continuous variables, while categorical variables were analyzed with the chi-square test or Fisher’s exact test. Intragroup comparisons over time employed repeated-measures analysis of variance for continuous variables. Changes within groups after six months were assessed using paired t-tests. All statistical analyses adhered to a significance level of p < 0.05.

## Results

Sociodemographic characteristics

**Figure 1:**
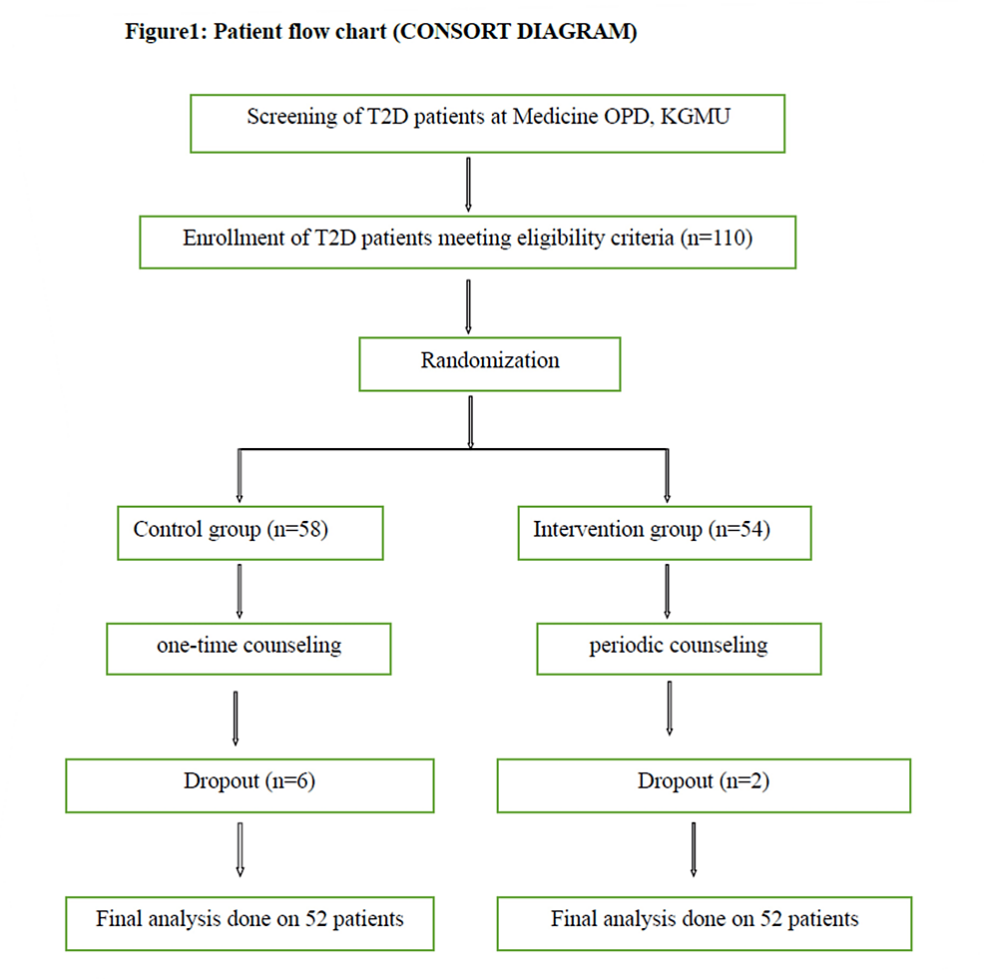
Patient flowchart (Consolidated Standards of Reporting Trials (CONSORT) diagram). T2D = type 2 diabetes; KGMU = King George’s Medical University; OPD = outpatient department

Figure 1 presents the Consolidated Standards of Reporting Trials (CONSORT) diagram. The baseline sociodemographic characteristics of the study participants were compared between the control and intervention groups, as presented in Table 1. The gender distribution revealed that 55.8% of males were in the control group, while 46.2% were in the intervention group. For females, 44.2% were in the control group, and 53.8% were in the intervention group. These proportions showed no statistically significant difference between the groups (p = 0.327). Similarly, there were no significant differences in religion, marital status, occupation, family type, and family history of diabetes between the two groups (p > 0.05). Education levels, however, exhibited a marginal difference (p = 0.074), with the intervention group having a higher proportion of postgraduate individuals (15.4%) compared to the control group (1.9%). The mean age was comparable between the control (48.24 ± 10.13 years) and intervention (50.87 ± 10.54 years) groups, with no statistically significant difference observed (p = 0.199). These findings suggest that, at baseline, the study groups were well-matched in terms of sociodemographic characteristics, minimizing potential confounding factors in subsequent analyses.

**Table 1:** Baseline sociodemographic characteristics of the study participants. Chi-square test for categorical variables (gender, religion, marital status, education, occupation, family type, family history of diabetes), and independent t-tests for a continuous variable (age).

Characteristics		Control (n = 52), N (%)	Intervention (n = 52), N (%)	P-value
Gender	Male	29 (55.8)	24 (46.2)	0.327
	Female	23(44.2)	28 (53.8)	
Religion	Hindu	42 (80.8)	37 (71.2)	0.251
	Muslim	10 (19.2)	15 (28.8)	
Marital status	Married	51 (98.1)	50 (96.2)	1.000
	Unmarried	1 (1.9)	2 (3.8)	
Education	High school	14 (26.9)	12 (23.1)	0.074
	Intermediate	13 (25.0)	15 (28.8)	
	Graduate	24 (46.2)	17 (32.7)	
	Postgraduate	1 (1.9)	8 (15.4)	
Occupation	Employed	23 (43.1)	18 (34.6)	0.375
	Unemployed	29 (56.9)	34 (65.4)	
Family type	Joint	28 (53.8)	29 (55.8)	0.844
	Nuclear	24 (46.2)	23 (44.2)	
Family history of diabetes	Yes	19 (36.5)	25 (48.1)	0.234
	No	33 (63.5)	27 (51.9)	
Age (mean ± SD) (in years)		48.24 ± 10.13	50.87 ± 10.54	0.199

Anthropometric measurements

Table 2 presents the baseline anthropometric measurements and the duration of diabetes among the study participants. The mean weight in the control group was 66.67 ± 11.51 kg, while in the intervention group, it was 67.14 ± 11.19 kg, with no statistically significant difference between the groups (p = 0.835). Likewise, the BMI in the control group (25.61 ± 4.09 kg/m²) and the intervention group (26.29 ± 6.01 kg/m²) showed no significant difference (p = 0.503). The weight-hip ratio exhibited a marginal difference (p = 0.144), with the control group having a mean of 0.88 ± 0.04 and the intervention group 0.89 ± 0.04. The duration of diabetes was comparable between the control (3.39 ± 3.48 years) and intervention (4.67 ± 6.19 years) groups, with no significant difference observed (p = 0.199). These findings indicate that, at baseline, there were no substantial differences in anthropometric measurements and the duration of diabetes between the control and intervention groups, ensuring a balanced starting point for the study intervention.

**Table 2:** Baseline anthropometry and duration of diabetes among the study participants. Independent t-tests for continuous variables (weight, body mass index, weight-hip ratio, duration of diabetes).

Variables		Mean ± SD	95% CI		P-value
			Lower	Upper	
Weight (kg)	Control	66.67 ± 11.51	-4.96	4.01	0.835
	Intervention	67.14 ± 11.19			
Body mass index (kg/m^2^)	Control	25.61 ± 4.09	-2.71	1.34	0.503
	Intervention	26.29 ± 6.01			
Weight-hip ratio	Control	0.88 ± 0.04	-0.026	0.004	0.144
	Intervention	0.89 ± 0.04			
Duration of diabetes (years)	Control	3.39 ± 3.48	-3.25	0.69	0.199
	Intervention	4.67 ± 6.19			

Clinical characteristics

The baseline clinical parameters of the study participants are presented in Table 3. For fasting blood sugar, postprandial blood sugar, HbA1c, systolic blood pressure (SBP), diastolic blood pressure (DBP), and Perceived Stress Scale (PSS), there were no significant differences between the control and intervention groups (p > 0.05). The mean fasting blood sugar levels were 171.96 ± 79.19 mg/dL in the control group and 193.09 ± 99.25 mg/dL in the intervention group. Similarly, the mean postprandial blood sugar levels were 249.28 ± 112.61 mg/dL in the control group and 243.77 ± 88.55 mg/dL in the intervention group. HbA1c levels were 8.31 ± 1.31% in the control group and 8.74 ± 2.03% in the intervention group. SBP was 134.84 ± 20.69 mmHg in the control group and 134.78 ± 15.18 mmHg in the intervention group. DBP was 87.33 ± 11.01 mmHg in the control group and 86.24 ± 12.12 mmHg in the intervention group. PSS scores were 31.54 ± 4.42 in the control group and 32.85 ± 3.42 in the intervention group. While some parameters showed trends toward significance (p < 0.1), no statistically significant differences were observed. This suggests that, at baseline, the clinical parameters were comparable between the control and intervention groups, providing a solid foundation for assessing the impact of the intervention over time.

**Table 3:** Baseline clinical parameters of the study participants. Independent t-tests for continuous variables (fasting blood sugar, postprandial blood sugar, glycosylated hemoglobin, systolic blood pressure, diastolic blood pressure), and Kolmogorov-Smirnov test for confirming normal distribution.

Parameters		Mean	95% CI		P-value
			Lower	Upper	
Fasting blood sugar (mg/dL)	Control	171.96 ± 79.19	-56.26	14.02	0.236
	Intervention	193.09 ± 99.25			
Postprandial blood sugar (mg/dL)	Control	249.28 ± 112.61	-34.21	45.22	0.785
	Intervention	243.77 ± 88.55			
Glycosylated hemoglobin (%)	Control	8.31 ± 1.31	-1.10	0.23	0.208
	Intervention	8.74 ± 2.03			
Systolic blood pressure (mmHg)	Control	134.84 ± 20.69	-7.07	7.19	0.987
	Intervention	134.78 ± 15.18			
Diastolic blood pressure (mmHg)	Control	87.33 ± 11.01	-3.45	5.65	0.633
	Intervention	86.24 ± 12.12			
Perceived Stress Scale (score range = 0–40)	Control	31.54 ± 4.42	-2.85	0.23	0.095
	Intervention	32.85 ± 3.42			

After the six-month follow-up

The comparison of blood pressure at the last follow-up between the control and intervention groups is presented in Table 4. After six months, the mean SBP in the control group was 132.15 ± 14.867 mmHg, and in the intervention group, it was 129.15 ± 9.123 mmHg. Although there was no statistically significant difference between the groups (p = 0.218), the control group showed a slightly higher mean SBP. Similarly, for DBP after six months, the control group had a mean of 85.80 ± 7.26 mmHg, while the intervention group had 83.67 ± 6.97 mmHg. The difference, though not statistically significant (p = 0.132), suggests a trend toward lower DBP in the intervention group. These findings indicate a potential positive impact of the intervention on blood pressure control, with a notable trend toward lower values in the intervention group at the last follow-up.

**Table 4:** Change in systolic and diastolic blood pressure in the intervention group at six months from the baseline. Independent t-tests for continuous variables (systolic blood pressure after six months and diastolic blood pressure after six months). *: A p-value less than 0.05 is significant. **: A p-value less than 0.01 is highly significant.

Blood Pressure (BP)		Mean ±SD	95% CI		P value
			Lower	Upper	
Systolic BP after 6 months (mmHg)	Control	132.15 ±14.867	-1.798	7.798	0.218
	Intervention	129.15 ±9.123			
Diastolic BP after 6 months (mmHg)	Control	85.80±7.26	-0.66	4.93	0.132
	Intervention	83.67±6.97			

Table 5 illustrates the change in SBP and DBP within the intervention group over six months compared to the baseline. The mean difference in SBP was 5.54 ± 9.77 mmHg, showing a statistically significant increase (p = 0.0001), indicating a positive change toward elevated SBP. Similarly, the change in DBP was 2.48 ± 7.78 mmHg, with a significant increase (p = 0.026), suggesting a noteworthy improvement in DBP. These results indicate that the intervention had a favorable impact on blood pressure management within the intervention group, with a substantial decrease in DBP and a notable increase in SBP over the six-month period. The findings emphasize the effectiveness of the intervention in achieving significant changes in blood pressure parameters, providing valuable insights into potential improvements in cardiovascular health.

**Table 5:** Change in systolic and diastolic blood pressure in the intervention group at six months from the baseline. Paired t-tests for continuous variables (change in systolic blood pressure after six months from the baseline and change in diastolic blood pressure after six months from the baseline). *: A p-value less than 0.05 is significant. **: A p-value less than 0.01 is highly significant.

Blood pressure	Mean difference ± SD	t value	P-value	95% CI	
				Lower	Upper
Change in systolic blood pressure after six months from the baseline (mmHg)	5.54 ± 9.77	4.09	0.0001**	2.82	8.26
Change in diastolic blood pressure after six months from the baseline (mmHg)	2.48 ± 7.78	2.301	0.026*	0.316	4.645

Table 6 presents the change in SBP and DBP within the control group after six months compared to the baseline. The mean difference in SBP was 2.308 ± 9.388 mmHg, demonstrating a modest increase that did not reach statistical significance (p = 0.082). Similarly, the change in DBP was 1.692 ± 6.717 mmHg, with a borderline increase that did not achieve statistical significance (p = 0.075). Although the observed changes in both SBP and DBP were not statistically significant, there was a trend toward an increase in blood pressure within the control group over the six-month period. These findings suggest a potential need for further investigation or consideration of additional interventions to address blood pressure management in the control group.

**Table 6:** Change in systolic and diastolic blood pressure in the control group after six months from the baseline. Paired t-tests for continuous variables (change in systolic blood pressure after six months from the baseline and change in diastolic blood pressure after six months from the baseline).

Blood pressure	Mean difference ± SD	t value	P-value	95% CI	
				lower	Upper
Change in systolic blood pressure after six months from the baseline (mmHg)	2.308 ± 9.388	1.773	0.082	-0.306	4.921
Change in diastolic blood pressure after six months from the baseline (mmHg)	1.692 ± 6.717	1.817	0.075	-1.568	4.817

## Discussion

The findings of this study provide valuable insights into the efficacy of one-time versus periodic lifestyle modification counseling in managing blood pressure among individuals with T2DM. Comparing the results of our study with previous research, we observe several important implications.

Baseline characteristics of the study participants demonstrated a well-matched distribution across sociodemographic, anthropometric, and clinical parameters in the control and intervention groups, minimizing potential confounding factors. This alignment is consistent with studies such as those by Emdin et al. [9] and Chen et al. [10], which emphasized the importance of balanced participant characteristics for robust intervention evaluations.

At the last follow-up, comparable mean SBP and DBP values were observed between the control and intervention groups. While not statistically significant, the intervention group showed slightly lower values, indicating a potential trend toward improved blood pressure control. These findings align with the results of studies such as those by Kumari et al. [11], which also reported modest improvements in blood pressure with lifestyle modification counseling.

Within the intervention group, the analysis of changes from baseline revealed a statistically significant reduction in DBP and a modest increase in SBP after six months. This finding contrasts with previous studies such as those by Wing [12] and Huang et al. [13] which reported significant improvements in both SBP and DBP with long-term lifestyle interventions. The observed increase in SBP in our study warrants careful consideration and underscores the need for ongoing monitoring and adjustment of counseling strategies, consistent with the findings of another study [14].

Furthermore, our study contributes to the broader discussion on integrating lifestyle modification counseling into routine clinical practice for T2DM management. While evidence supporting the efficacy of lifestyle interventions continues accumulating, challenges such as limited access to counseling services and patient adherence remain significant barriers. Future research efforts should address these challenges and develop scalable interventions that can be effectively implemented in diverse healthcare settings.

Limitations of the study

The limitations of the study include a relatively small sample size (110 participants), which may limit the generalizability of the findings to broader populations of individuals with T2DM. Additionally, the study’s duration of six months may not capture the longer-term effects of lifestyle modification counseling on blood pressure control in T2DM patients. Furthermore, the study’s focus on blood pressure as the primary outcome measure may overlook other important health indicators affected by lifestyle modifications in T2DM management. Lastly, while efforts were made to minimize confounding factors through matching of sociodemographic and clinical parameters at baseline, the potential for uncontrolled confounders influencing the results cannot be entirely ruled out.

## Conclusions

The results suggest that periodic counseling could positively influence DBP, while the increase in SBP indicates the need for further research. Future studies should consider personalized counseling methods and examine the specific impacts of lifestyle changes on different blood pressure components in T2DM.
